# A Comparative Assessment of the Antifibrotic Effect of Nintedanib Administered via a Medicated Diet or Oral Gavage in a Rat Model of Bleomycin-Induced Pulmonary Fibrosis

**DOI:** 10.3390/ijms27188158

**Published:** 2026-09-13

**Authors:** Vanessa Pitozzi, Paola Caruso, Silvia Pontis, Francesca Ruscitti, Maria Gloria Pittelli, Giancarlo Aquino, Roberta Volta, Alice Pappani, Mariarosaria Barrea, Federico Quaini, Costanza Anna Maria Lagrasta, Antonella Maria Nogara, Paolo Spagnolo, Marcello Trevisani

**Affiliations:** 1 Chiesi Farmaceutici S.p.A., Global Research and Preclinical Development, Largo Belloli, 11/A, 43122 Parma, Italys.pontis@chiesi.com (S.P.); f.ruscitti@chiesi.com (F.R.); m.pittelli@chiesi.com (M.G.P.); g.aquino@chiesi.com (G.A.); r.volta@chiesi.com (R.V.); a.pappani@chiesi.com (A.P.); mariarosaria.barrea@gmail.com (M.B.); m.trevisani@chiesi.com (M.T.); 2 Department of Medicine and Surgery, University of Parma, 43126 Parma, Italy; federico.quaini@unipr.it (F.Q.); costanzaannamaria.lagrasta@unipr.it (C.A.M.L.); antonellamaria.nogara@unipr.it (A.M.N.); 3 Respiratory Disease Unit, Department of Cardiac Thoracic, Vascular Sciences and Public Health, University of Padova, 35128 Padova, Italy; paolo.spagnolo@unipd.it

**Keywords:** nintedanib, bleomycin, medicated diet, target engagement, biomarkers

## Abstract

Idiopathic pulmonary fibrosis (IPF) remains a progressive and fatal disease despite major advances in antifibrotic therapy. Pirfenidone and nintedanib slow lung function decline, and the PDE4B inhibitor nerandomilast and treprostinil have recently shown promise as next-generation treatments. However, current therapies neither halt nor reverse disease progression, and tolerability issues often limit adherence. We compared the antifibrotic activity and plasma levels of nintedanib administered by either oral gavage or dietary supplementation in a rat model of pulmonary fibrosis. Male Sprague-Dawley rats received intratracheal bleomycin (1 U/kg) on days 0 and 4. From day 7 to day 28, animals were treated with nintedanib (100 mg/kg/day) by either oral gavage or medicated chow. Lung weight, fibrosis biomarkers (procollagen-I, metalloproteinase-7 or MMP7, WNT1-inducible signaling pathway protein or WISP-1), epithelial injury marker KL-6, target engagement biomarkers (Fibroblast Growth Factor 2 or FGF-2, Vascular Endothelial Growth Factor or VEGF), plasma drug levels, and histological fibrosis scores were evaluated. Both administration regimens significantly reduced procollagen-I, WISP-1 and KL-6, and histological fibrosis scores. Oral gavage produced approximately fourfold-higher peak plasma concentrations of nintedanib 30 min after dosing, whereas dietary administration resulted in lower, more stable plasma levels with reduced variability while resulting in comparable antifibrotic efficacy. In conclusion, nintedanib retained robust antifibrotic activity when administered via dietary supplementation despite lower, but continuous, active plasma concentrations. Collectively, these findings indicate that optimizing drug delivery and absorption kinetics may achieve sustained therapeutic efficacy while reducing unnecessary exposure to supratherapeutic concentrations, thereby potentially improving tolerability.

## 1. Introduction

Idiopathic pulmonary fibrosis (IPF) is a progressive, fibrosing interstitial lung disease of unknown etiology that leads to progressive and irreversible decline in lung function, poor quality of life, and ultimately respiratory failure. The disease predominantly affects older adults and is associated with a poor prognosis, with a median survival of 3–5 years from diagnosis, if untreated [1,2]. Over the past decade, therapeutic advances have led to the approval of antifibrotic agents capable of slowing disease progression. Current management for IPF, recommended by different guidelines [1], consists of continuous long-term treatment with antifibrotic agents requiring daily administration and the ongoing monitoring of tolerability and disease progression. Pirfenidone exerts both antifibrotic and anti-inflammatory effects, although its mechanism of action is not fully defined. In patients with IPF, pirfenidone, at a maintenance dose of 801 mg three times daily (2403 mg/day), has been shown to slow FVC loss and reduce the risk of disease progression [3]. Similarly, nintedanib, a multi-target tyrosine kinase inhibitor acting on Platelet-Derived Growth Factor (PDGF), FGF, and VEGF receptor pathways, at 150 mg twice daily (300 mg/day), attenuates FVC decline [4]. More recently, nerandomilast, a preferential phosphodiesterase 4B (PDE4B) inhibitor (oral 18 mg twice daily), and treprostinil, a prostacyclin analog (inhaled titration from 18 μg to a target dose of 72 μg four times daily), have proven efficacious in slowing down functional decline and disease progression in patients with IPF by targeting several inflammatory and fibrotic pathways [5,6,7,8]. Despite these advances, IPF remains an incurable disease, highlighting the ongoing need for an improved understanding of disease pathobiology and the development of more effective therapeutic strategies. Overall, these therapies are generally well tolerated; however, their use could be associated with manageable but clinically relevant adverse events, including gastrointestinal disturbances, liver enzyme elevations, and photosensitivity, which may impact treatment adherence and require dose adjustment or supportive management [5,9,10,11].

We recently demonstrated that orally administered nintedanib reduced fibrotic outcomes and modulated biomarkers associated with its mechanism of action in a model of bleomycin-induced lung fibrosis in a rat. Notably, we identified VEGF as a potential pharmacodynamic marker of target engagement, supporting its translational relevance for clinical application [12]. In the present study, we aimed to further characterize the in vivo antifibrotic profile of nintedanib under two different administration regimens: oral gavage and dietary (chow) supplementation. We assessed nintedanib plasma levels and used VEGF as evidence of target engagement, confirming effective VEGF receptor modulation under both administration regimens. Additionally, archetypal tissue biomarkers of fibrosis (procollagen I, MMP7, and WISP-1), biomarkers linked to epithelial injury (KL-6) and nintedanib’s mechanism of action (FGF2) were assessed. Automated histological analyses of lung fibrosis and Ashcroft scores were also undertaken. This study sought to evaluate whether sustained but lower plasma concentrations of nintedanib, achieved through dietary administration, were sufficient to retain antifibrotic activity comparable to that obtained with conventional oral gavage.

## 2. Results

### 2.1. Nintedanib Quantification in Chow

Before in vivo administration, medicated diet was analyzed by Liquid Chromatography–Mass Spectrometry (LC–MS; chromatograms are shown in Supplementary Figure S1). A total of 14 pellets were analyzed, and the percentage of active pharmaceutical ingredient (API, nintedanib, % *w*/*w*) and RSD% (% relative standard deviation) were evaluated to obtain content and content uniformity estimations. The nominal content of nintedanib in the medicated diet was 0.16% *w*/*w* (1.6 g/kg, Table 1). The analyses revealed a drug content consistent with the nominal concentration (mean recovery: 102%) and satisfactory content uniformity (RSD < 10%), indicating adequate formulation homogeneity and supporting its use for in vivo studies.

While these data demonstrated the integrity of the API in this type of product, preliminary stability studies on nintedanib “as is” were conducted: storage at 40 °C for 48 h. The aim was to simulate the manufacturing conditions of medicated food. 

### 2.2. Bleomycin-Induced Lung Fibrosis: Feasibility Study on Food Intake

**Figure 1:**
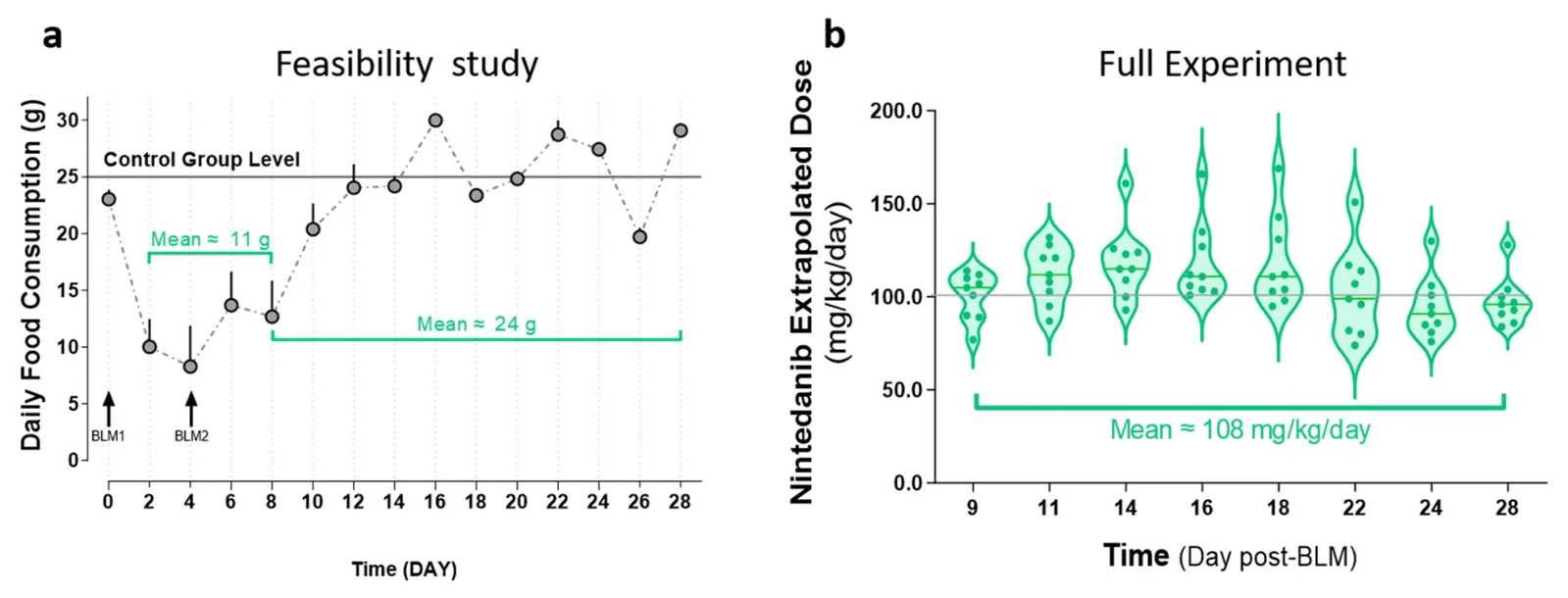
Food consumption assessment. (**a**) Feasibility study: Food consumption, expressed as grams of chow, measured every other day from day 0 to day 28 in BLM-treated rats. Individual values are shown (gray circles, *n* = 9). The solid horizontal gray line indicates the mean food consumption of control animals (not treated with BLM, *n* = 3), whereas the two solid horizontal green lines represent the mean daily chow intake of BLM-treated rats during the acute (days 0–7) and late post-BLM phases (days 8–28) respectively. (**b**) Main BLM experiment: Predicted nintedanib intake based on chow consumption measured from day 9 (+2 day post-medicated chow administration) to day 28. Individual values are shown (green circles, *n* = 9). Solid horizontal green lines represent the average nintedanib intake for each day. The solid gray line represents the intended target dose of nintedanib (100 mg/kg).

During the feasibility study, the measured daily food intake was approximately 24 g per animal in the control (no-BLM) over the whole observation period (days 0–28) and in BLM-treated animals (from day 8 to 28, Figure 1a). In contrast, BLM-treated rats showed a markedly reduced food intake during the “acute” post-BLM phase (approximately 11 g/day, days 0–7), likely due to the proximity to the double BLM administration (Figure 1a). Considering that the nintedanib dosing period under the therapeutic regimen in the main study spans days 7–28, a mean daily food consumption of 24 g per rat was assumed for dose calculations and medicated chow preparation, as supported by the data shown in Figure 1a. Based on these calculations, detailed in the Section 4, a final concentration of 1.6 g of nintedanib per kg of chow was selected as the optimal one to ensure that animals consistently received an estimated target dose of approximately 100 mg/kg/day (Figure 1a). The adequacy of the above chow formulation was further confirmed during the main study analysis on chow consumption (Figure 1b). Indeed, food intake in the medicated diet group remained within the expected range, providing an estimated nintedanib average intake of approximately 108 mg/kg/day (Figure 1b), indicating that the selected dietary concentration was suitable to sustain the intended daily nintedanib dose throughout the therapeutic window. To provide a more complete assessment of individual animal drug intake, the estimated dose received by each rat at each monitoring checkpoint is reported in Supplementary Table S4.

### 2.3. Bleomycin-Induced Lung Fibrosis: Main Experiment

#### 2.3.1. Animals’ General Health Condition, Body Weight and Nintedanib Food Intake Calculation

**Figure 2:**
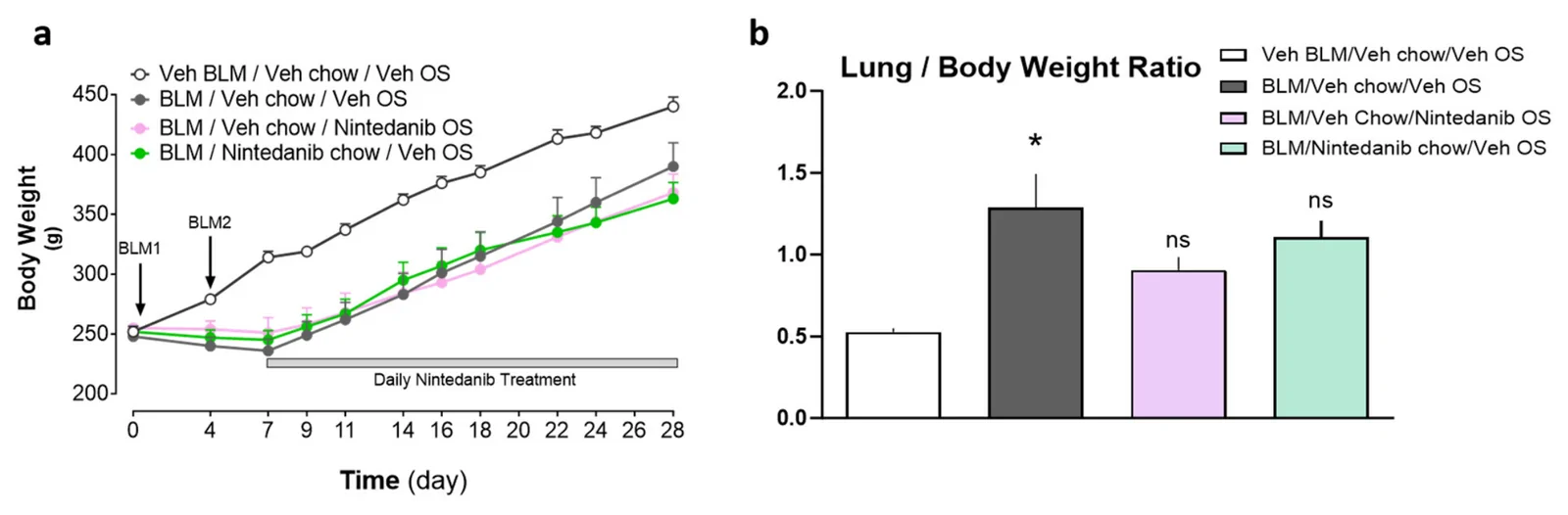
The experimental protocol. (**a**) Changes in body weight during the entire experimental protocol. Rats were challenged intratracheally with BLM (1 U/kg) on day 0 and day 4. Beginning on day 7, nintedanib was administered orally (100 mg/kg, pink circles and lines) or through a medicated diet (100 mg/kg, green circles and lines) until day 28. Each symbol represents the group mean at the indicated time point. (**b**) The lung/body weight ratio in control and BLM-treated animals. Data are presented as the mean ± SEM; *n* = 9 animals per group. Statistical analysis was performed with a one-way ANOVA followed by Dunnett’s test. * *p* < 0.05 versus the control group (white column); ns, not statistically significant versus the BLM + vehicle group (dark gray column).

No mortality occurred during the entire study, and all animals reached the endpoint on day 28. BLM-exposed animals displayed a transient impairment in body weight progression compared to control rats, with the greatest divergence observed on day 7 (Figure 2a). Body weight curves were comparable among BLM-treated groups irrespective of nintedanib treatment or its vehicle, with no evidence of an additional treatment-related effect on body weight (Figure 2a).

Exposure to BLM was also associated with a significant elevation in the lung/body weight ratio compared to control animals, consistent with the combined contribution of pulmonary inflammation, fluid retention, extracellular matrix accumulation, and fibrotic tissue remodeling. The administration of nintedanib, either by oral gavage or via a medicated diet, resulted in an approximately 50% reduction in the BLM-induced increase in lung weight. Although a clear trend toward attenuation was observed in both treatment groups, statistical significance was not achieved (Figure 2b). Nintedanib administered by medicated diet (1.6 g/kg chow) was confirmed to be an appropriate formulation to ensure consistent drug intake, enabling animals to reliably achieve a calculated target dose of approximately 100 mg/kg/day (Figure 1b). These data were further confirmed by target engagement analysis (see next sections).

#### 2.3.2. Histological Analysis

**Figure 3:**
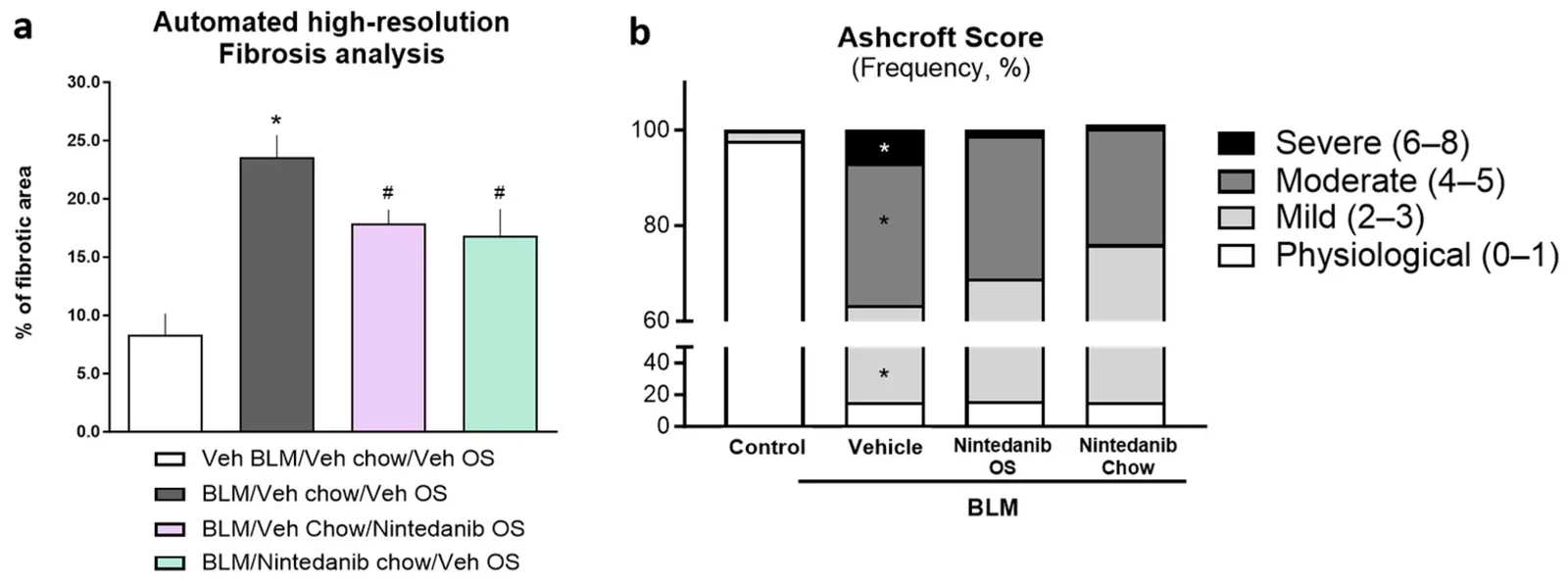
An evaluation of the antifibrotic activity of nintedanib: (**a**) The inhibitory effect of nintedanib administered by oral gavage (light pink columns) or by a medicated diet (light green column) on automated lung fibrosis analysis vs. the BLM group (dark gray column). (**b**) The effect of nintedanib on Ashcroft scores, measured on Masson’s trichrome-stained slides, relative to animals treated with BLM only. Data are presented as the mean ± SEM; *n* = 9 animals per group. Statistical analysis was performed with a one-way ANOVA followed by Dunnett’s test. * *p* < 0.05 versus control; # *p* < 0.05 versus BLM + vehicle; a one-way ANOVA followed by Dunnett’s multiple-comparison test.

BLM administration induced a statistically significant increase in the area affected by fibrotic lesions, as quantified by automated image analysis (Figure 3a). Ashcroft scores (Figure 3b) were stratified into four categories according to the extent of fibrosis, from 0 to 1 (physiological), 2 to 3 (mild), 4 to 5 (moderate), and 6 to 8 (severe), and expressed as the frequency of each severity class across treatment groups. Compared with the control group, the BLM group showed a significantly higher overall frequency of fibrotic lesions, with an increase of 15% across severity categories (Figure 3a). Nintedanib administered either by oral gavage or through the medicated diet significantly shifted the distribution toward milder scores compared to the untreated BLM group, increasing the frequency of mild fibrosis by 5% (oral gavage) and by 13% (medicated diet) while reducing the frequency of moderate-to-severe fibrosis in both groups (Figure 3b).

#### 2.3.3. Tissue Biomarker Analysis

**Figure 4:**
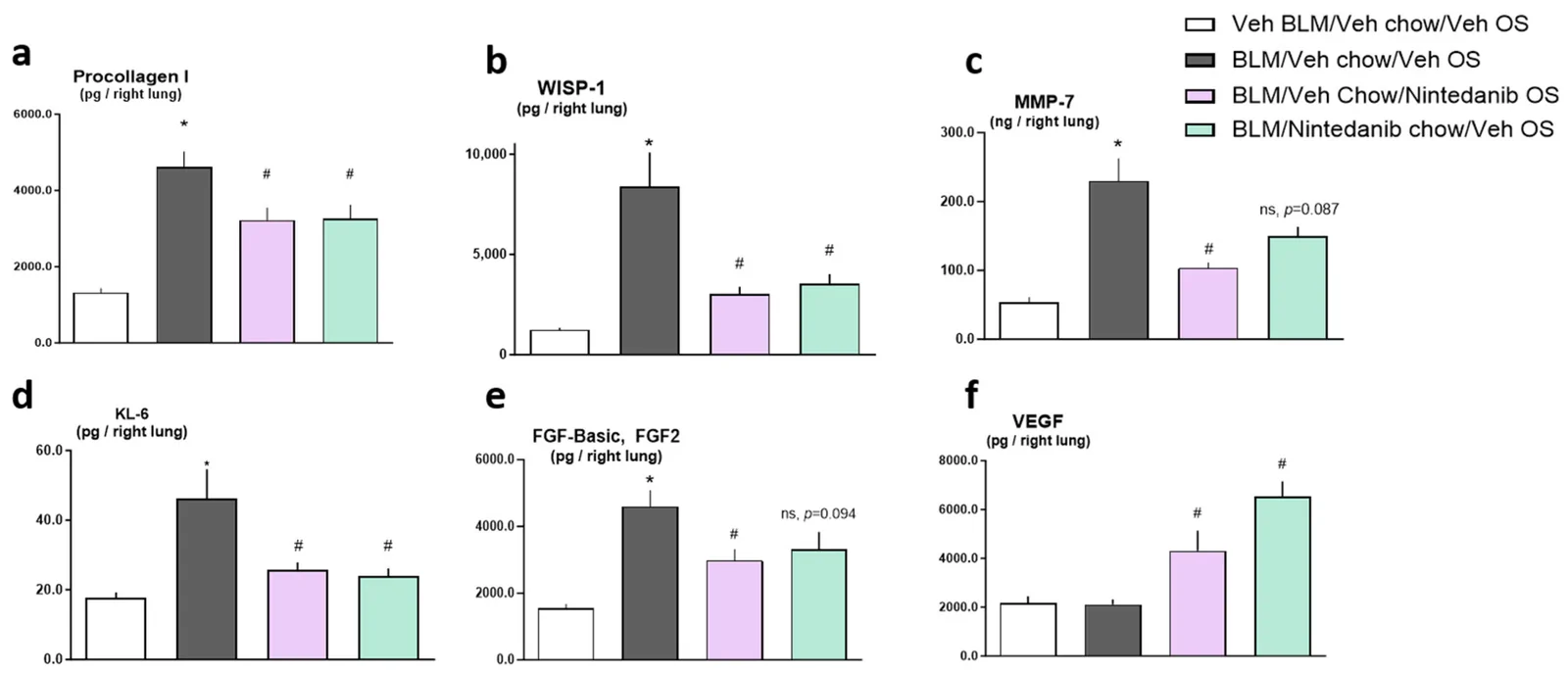
Evaluation of effect of nintedanib administered by oral gavage (light pink columns) or by medicated diet (light green columns) against multiple fibrotic markers. (**a**) Procollagen I; (**b**) WISP1; (**c**) MMP-7; (**d**) KL-6; (**e**) FGF2; (**f**) VEGF quantified by ELISA in lung homogenate supernatants compared to BLM group (dark gray columns). Data are presented as mean ± SEM; *n* = 9 animals per group. Statistical analysis was performed using one-way ANOVA followed by Dunnett’s multiple-comparison test. * *p* < 0.05 versus control; # *p* < 0.05 versus BLM + vehicle (dark gray column), ns, not statistically significant versus the BLM + vehicle group (dark gray column).

To further characterize the antifibrotic activity of nintedanib under the two administration regimens, relevant tissue biomarkers related to extracellular matrix remodeling, epithelial injury, and target engagement were quantified in lung homogenates. BLM administration significantly increased fibrosis-associated biomarkers, including procollagen-I, WISP-1 and MMP7, together with the epithelial injury marker Mucin 1 or Krebs von den Lungen 6 (MUC1/KL-6) and FGF2, confirming the establishment of lung injury and fibrotic remodeling. Nintedanib, administered either by oral gavage or through the medicated diet, reduced the BLM-induced rise in these biomarkers, with an overall comparable profile between the two dosing approaches. Specifically, nintedanib exhibited a significant inhibitory effect on WISP1 (75% and 67% reduction) and KL-6 (72% and 78% reduction) following oral gavage and medicated diet, respectively (Figure 4a–d). In line with its mechanism of action, both nintedanib administration approaches also modulated soluble mediators linked to target engagement, with changes in FGF2 and significantly increased VEGF levels (Figure 4e,f), consistent with VEGFR pathway inhibition and supporting the pharmacodynamic modulation of nintedanib-related pathways [12].

#### 2.3.4. Nintedanib Plasma Levels

**Figure 5:**
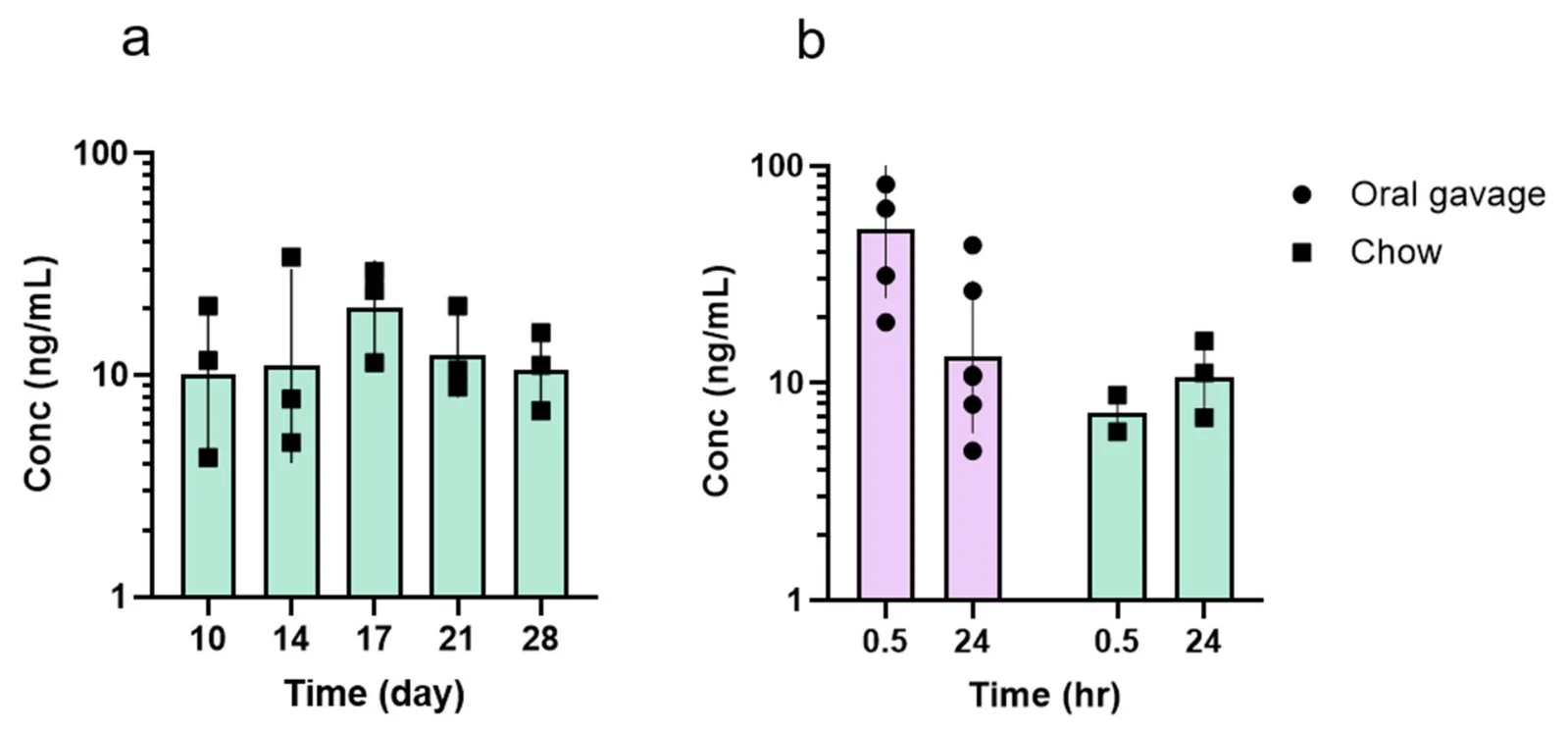
An assessment of nintedanib plasma concentrations following administration via medicated diet (square dots; light green columns) or by oral gavage (circle dots; light pink columns). (**a**) Individual and geometric mean (±geometric SD) plasma concentrations from rats receiving nintedanib through a medicated diet (square dots—light green columns; *n* = 3 animals) were collected to evaluate plasma concentrations across this study, on days 10, 14, 17, 21, and 28, using a consistent early-morning sampling time point. (**b**) Individual and geometric mean (±geometric SD) plasma concentrations from rats administered with nintedanib by oral gavage at 100 mg/kg once daily (circle dots—light pink columns, *n* = 4–5 animals) were analyzed on the final day of treatment (day 28) at two time points (0.5 and 24 h) post-dose. These values were directly compared with those obtained from the medicated diet group (square dots—light green columns, *n* = 2–3 animals) sampled at the same clock times as those in the oral gavage group.

Plasma samples from BLM-treated rats receiving nintedanib through a medicated diet were collected at 9 a.m. on days 10, 14, 17, 21, and 28 to evaluate plasma concentrations across this study, using a consistent early-morning sampling time point (N = 3 for each time point, Figure 5a). On day 28, animals treated with a medicated diet were also compared with animals treated with oral gavage at two distant time points (0.5 and 24 h); to allow for a direct temporal comparison across treatment modalities, animals in the chow groups were sampled at the same clock times as those in the oral gavage group, acknowledging that these time points do not represent defined pharmacokinetic intervals relative to drug intake. Nintedanib concentrations measured 30 min after oral gavage were markedly higher (approximately fourfold, Figure 5b) than those observed in the medicated diet, which instead showed lower and less variable plasma concentrations (10–20 ng/mL; Figure 5a,b).

## 3. Discussion

Pirfenidone and nintedanib are established antifibrotic treatments for IPF based on their ability to attenuate lung functional decline and delay disease progression. Nerandomilast and, more recently, inhaled treprostinil are also associated with a smaller decline in FVC than placebo in patients with IPF [3,4,5,6,7,8,13]. Despite these advances, current treatment options do not offer a definitive cure for IPF, leaving a substantial unmet medical need for therapies capable of inducing true disease remission or tissue repair/regeneration. Moreover, beyond their limited ability to modify the natural history of the disease, existing antifibrotic treatments are often associated with a substantial burden of adverse events, which can negatively impact patient compliance and long-term adherence. Specifically, pirfenidone is commonly associated with gastrointestinal disturbances, photosensitivity, and fatigue [10,14], whereas nintedanib may cause diarrhea, hepatic enzyme elevations, decreased appetite and weight loss [15]. These tolerability issues can lead to dose reductions or the temporary or permanent discontinuation of therapy, ultimately limiting real-world effectiveness [16,17,18]. Although nerandomilast has been designed to improve tolerability within the PDE4 inhibitor class, longer-term data are still required to fully establish its safety profile and clinical positioning [19]. Lastly, inhaled treprostinil may facilitate increased drug deposition at the alveolar interface and could potentially be associated with a lower risk of systemic adverse effects. Yet, in the TETON-1 and -2 trials, one-third of patients discontinued treprostinil mainly due to adverse events [6,7]. Whether the adverse event profile could be further mitigated through a more accurate investigation of temporal drug exposure levels remains unclear. Continued efforts are ongoing to better understand IPF pathobiology, identify predictive biomarkers, and optimize pharmacokinetic/pharmacodynamic relationships. Current and future therapeutic strategies may benefit from a shift toward earlier intervention, sustained pathway modulation, and improved tolerability, with the aim of achieving more durable clinical benefit and, ultimately, altering the course of this devastating disease.

Preclinical animal models remain crucial for therapeutic target identification and validation and for assessing the efficacy of antifibrotic therapies. Among them, BLM-induced lung fibrosis in rodents is widely used because it reproduces several key features of IPF (e.g., ECM deposition) and has supported the preclinical evaluation of approved antifibrotic drugs. In the present investigation, lung fibrosis was induced in rats by double intratracheal BLM administration, a regimen intended to model repeated epithelial injury more closely than a single fibrotic insult [20]. Using this therapeutic setting, we compared nintedanib delivered by conventional oral gavage or through a medicated diet.

Our study demonstrates that nintedanib retains similar antifibrotic efficacy when administered via dietary supplementation compared to conventional oral gavage in a rat model of BLM-induced fibrosis. Specifically, nintedanib, whether administered by oral gavage or via medicated chow, elicited comparable antifibrotic effects, as evidenced by reductions in lung markers of fibrosis (procollagen I, MMP7, and WISP-1, Figure 4a–c) and biomarkers linked to nintedanib’s mechanism of action (FGF2, Figure 4e) and to epithelial injury (KL-6, [21], Figure 4d). Moreover, the antifibrotic effect measured by the Ashcroft score and automated high-resolution fibrosis quantification were comparable under the two administration regimens (Figure 3). Interestingly, dietary administration resulted in lower nintedanib plasma levels (approximately fourfold reduction, Figure 5) compared with the early and transient peak plasma concentrations observed after oral gavage administration while still achieving an adequate target engagement consistent with VEGFR pathway inhibition, as evidenced by a significant increase in VEGF levels [12] (Figure 4f). These findings have important implications for identifying the compound plasma levels required to sustain target engagement, pharmacodynamic effects, and efficacy, suggesting that transient supratherapeutic concentrations may not be necessary to achieve therapeutic benefit.

Systemic exposure following the repeated oral administration of nintedanib in rats has been extensively characterized in a previous study from our group [12]. The plasma concentrations measured on the final day of treatment showed that levels at 24 h were comparable to those considered clinically efficacious [22]. In contrast, concentrations at earlier time points substantially exceeded those required for target engagement and therapeutic activity, potentially contributing to undesired side effects. This pharmacokinetic profile is consistent with oral gavage administration, which delivers a defined bolus dose and typically results in pronounced peak plasma concentrations. In the present experiment, we showed that dietary administration resulted in a more constant drug intake, characterized by lower levels, comparable to those considered efficacious in the clinical setting [22] and reduced peak-to-trough variability (Figure 5a), in contrast to oral gavage which instead confirmed higher peak plasma concentrations (Figure 5b). From a pharmacokinetic perspective, despite the inherently variable and less controlled nature of intake, in-diet administration seems to result in sufficient plasma concentrations able to increased VEGF levels, as shown in Figure 4f. These data indicate that the antifibrotic efficacy of nintedanib might not be strictly dependent on high peak-mediated transient exposure but rather on sustained lower drug levels over time. This is consistent with its pharmacological profile as a multi-target tyrosine kinase inhibitor, modulating chronic signaling pathways involved in fibrogenesis.

The ability of nintedanib, administered as a medicated diet, to attenuate tissue markers and fibrosis without the need for “pulsatile” exposure suggests that the steady-state suppression of fibroblast activation, proliferation, and extracellular matrix deposition is sufficient to interfere with lung fibrosis establishment and recovery. Consequently, the observed antifibrotic effects indicate that, in this preclinical model, nintedanib administered via a medicated diet maintains sustained and sufficient suprathreshold levels to ensure sustained pharmacological coverage, without the peak-related exposures typically associated with oral gavage dosing.

From a preclinical and experimental standpoint, dietary delivery offers several advantages. It minimizes the need for repeated gavage procedures, thereby reducing animal stress and the potential for procedure-related confounding effects on inflammatory or fibrotic endpoints. This is particularly relevant in chronic models, where repeated handling may influence disease progression through stress-mediated pathways. In addition, dietary administration facilitates long-term studies with more physiologically relevant exposure profiles.

These concepts are supported by previous work with saracatinib/AZD0530, where incorporation into the diet was shown to provide a feasible and stable alternative to repeated oral dosing in chronic rat studies while reducing repeated handling and procedure-related stress. Consistent with this concept, our data indicate that medicated diet can sustain sufficient nintedanib exposure to preserve target engagement and antifibrotic efficacy, supporting this route as a valuable strategy for chronic preclinical fibrosis studies [23].

The finding of maintained efficacy under medicated diet administration also has implications for the therapeutic window of nintedanib. The observation that in vivo pharmacodynamic activity and efficacy with nintedanib can be achieved at lower and more stable plasma concentrations suggests a flexible exposure–response relationship and points toward a potentially broader therapeutic window. This could be associated with improved tolerability, as high peak exposures are often linked to adverse effects. Thus, sustained exposure at moderate levels of nintedanib may represent an optimal balance between efficacy and safety.

A potential limitation of this study design is the variability introduced by inter-individual differences in food intake, which necessitates careful monitoring and suitable normalization strategies. To mitigate this aspect, we conducted a pilot study to carefully quantify chow intake in BLM-treated rats across different post-treatment phases (Figure 1a). Based on these pilot results, 1.6 g per kg of chow was identified as the optimal amount of nintedanib to ensure animals consistently received a target dose of 100 mg/kg/day with the diet. This strategy tends to stabilize nintedanib exposure (dose assumption) throughout the entire experimental period, as shown in Figure 1a,b and the PK results (Figure 5a,b). Another limitation of the current study is that animals, housed under a 12/12 h light/dark cycle, may exhibit differential feeding behavior across the light and dark phases, which was not systematically monitored. Nevertheless, we assumed that food consumption in group-housed rats occurs not exclusively during the dark phase (night) but also during the light phase, as supported by our scientists’ direct observations indicating continued feeding during the light period. Furthermore, the intake of the medicated diet is likely to occur with a temporal pattern that differs from single oral gavage, in terms of both the rate of ingestion and kinetics of absorption.

More broadly, these findings have strategic implications for antifibrotic drug development. They reinforce the positioning of nintedanib as a robust benchmark compound in preclinical fibrosis models and highlight the importance of considering not only the dose but also the mode and temporal pattern of drug administration. The data might support a shift in focus toward therapeutic strategies that prioritize the continuous modulation of pathogenic pathways, rather than peak-driven pharmacodynamics. This principle may be particularly relevant for next-generation antifibrotic agents, where optimizing exposure profiles to achieve sustained target engagement could enhance both efficacy and tolerability.

In conclusion, our findings demonstrate that nintedanib maintains antifibrotic efficacy when delivered via medicated diet, despite lower and more stable drug plasma levels compared with oral gavage. While direct clinical extrapolations cannot be made from these findings, the results raise the possibility that sustained therapeutic efficacy may be achieved at exposure levels that are both clinically relevant and potentially better tolerated, warranting further investigation in translational medicine.

## 4. Materials and Methods

### 4.1. Animals

Male Sprague-Dawley (SD) rats, weighing 250–300 g, were obtained from Charles River Laboratories (Calco, Italy), housed individually and maintained with ad libitum access to food and water. All the experimental procedures involving animals were approved by the local ethics committees and authorized by the Italian Ministry of Health (authorization number: 1066/2015; approval date: 5 October 2015, and 246/2021-PR; approval date: 7 April 2021). Moreover, all in vivo activities were conducted in an AAALAC (Association for Assessment and Accreditation of Laboratory Animal Care, https://www.aaalac.org/ accessed on 11 August 2026)-accredited animal facility and in accordance with the European ethics standards and with Directive 2010/63/EU, Italian D. Lgs 26/2014; the revised “Guide for the Care and Use of Laboratory Animals” (Guide for the Care and Use of Laboratory Animals, 1996); and the ARRIVE guidelines (Animal Research: Reporting of In Vivo Experiments).

### 4.2. Medicated Diet Preparation and Nintedanib Quantification

Medicated chow containing nintedanib was custom-prepared by Mucedola (https://www.mucedola.eu/, accessed on 11 August 2026, Settimo Milanese, Italy) by incorporating the compound into a standard rodent diet. For nintedanib quantification, individual pellets were extracted with 10 mL of acetonitrile/water (60/40) and sonicated for 30 min, with vortex mixing conducted every 5 min. Then pellets were manually disintegrated with a spatula and again sonicated for 1 h. After 5 h, pellets were finally filtered through 0.22 µm Polytetrafluoroethylene (PTFE) filters and diluted 1/1000 with solvent for the analysis. Drug quantification was performed by an LC/MS-MRM experiment (see Supplementary Methods). Calibration and verification standards were prepared at about 0.5 µg/mL in the same solvent. Preliminary stability studies on nintedanib “as is” (40 °C for 48 h), performed to simulate medicated diet manufacturing conditions, were also conducted (see Section 2). Detailed LC–MS/MRM conditions and chromatograms are reported in the Supplementary Materials (Tables S1–S3 and Figure S1).

### 4.3. Bleomycin-Induced Lung Fibrosis

#### 4.3.1. Feasibility Study on Food Intake

To determine the appropriate amount of nintedanib to be incorporated into the rat diet to achieve a target dose of 100 mg/kg/day, we first quantified the mean daily chow consumption in two parallel groups: 3 control (BLM-untreated) and 9 BLM-treated animals. In this first study, individual food intake per rat was monitored every other day (from day 0 to day 28), enabling the accurate estimation of the nintedanib concentration required in the chow to ensure appropriate target dose delivery.

The appropriate concentration of nintedanib to be incorporated into chow was calculated as follows. The average daily food intake was approximately 24 g per rat (Figure 1a). A mean body weight of 350 g was considered appropriate to account for anticipated weight gain during this study. Thus, a target dose of 100 mg/kg/day for a rat weighing 350 g corresponds to 35 mg of nintedanib per day. To achieve this daily exposure via chow, 35 mg of nintedanib would be incorporated within 24 g of food, equivalent to a concentration of approximately 1.458 g of nintedanib in 1 kg of chow. To ensure consistent exposure above the target dose and to account for inter-animal variability in food intake and maintain a suprathreshold dosing regimen, a 10% overage (“buffer”) was applied, and the final concentration was set at 1.6 g of nintedanib for kg chow.

#### 4.3.2. Main Study

Thirty-six SD rats were used in the main study. Following acclimatization, rats were placed under light sevoflurane anesthesia and received the double intratracheal administration of BLM (bleomycin sulphate, Sigma-Aldrich, Burlington, MA, USA, 1 U/kg, *n* = 27) on day 0 and day 4 (Figure 2a) or 0.9% saline solution (Veh, *n* = 9) using a Penn Century Microsprayer (Penn-Century Inc., Wyndmoor, PA, USA) [12]. Seven days after the first BLM administration, BLM-treated rats were randomly allocated to three groups of 9 animals, with no significant differences in mean baseline body weight, receiving vehicle, nintedanib by oral gavage (100 mg/kg), or nintedanib through medicated chow from day 7 until the end of the experiment (day 28). To standardize experimental conditions across all groups, all animals received two intratracheal administrations (BLM or its vehicle) and daily oral gavage (nintedanib or its vehicle). Body weight was monitored as an indicator of overall health condition. Animals were sacrificed on day 28 by an intraperitoneal injection of 200 mg/kg thiopental (pentothal sodium, MSD Animal Health Srl, Milan, Italy). Whole lungs were removed and weighed, and right and left lobes were separated for the following histological and biochemical analyses. Plasma samples were obtained by blood collection from cardiac puncture and following centrifugation at 2000 *g* and 4 °C for 10 min, and aliquots were collected for subsequent biomarker evaluation and pharmacokinetic (PK) analysis. All samples were coded/blinded until the raw data were obtained. In the medicated diet group, chow consumption was monitored three times per week to ensure that animals received the intended dietary intake, as estimated during the pilot study.

### 4.4. Assessment of Nintedanib Plasma Levels

The bioanalysis and quantification of nintedanib in rat specimens were performed as already described in [12]. Briefly, plasma samples were extracted by means of protein precipitation with acetonitrile; the supernatant was directly injected into the LC/MS/MS system and analyzed using a fit-for-purpose bioanalytical method developed internally.

Plasma samples from BLM-treated rats receiving nintedanib through a medicated diet were collected at 9 a.m. on days 10, 14, 17, 21, and 28 to evaluate plasma concentrations across this study, using a consistent early-morning sampling time point (N = 2–3 for each time point). Plasma samples from rats administered nintedanib by oral gavage were analyzed on the final day of treatment (day 28, *n* = 4–5) at two time points (0.5 and 24 h) post-dose; for comparison, animals in the chow groups were sampled on the final day of treatment at the same clock times as those in the oral gavage group.

### 4.5. Histological Assessment

Left lung lobes from each animal were fixed in formalin and embedded in paraffin. Longitudinal sections (5 μm thickness) were obtained from each sample. Fibrotic lesions were evaluated on Masson’s trichrome-stained sections using a semiquantitative scoring method based on the Ashcroft scale (grades 0–8), as originally described by Ashcroft et al. [24] and subsequently modified by Hübner et al. [25]. Fibrosis was determined by light microscopy (ZEISS 433048, Oberkochen, Germany) at 100× magnification using a grid encompassing a tissue area of 0.23 mm^2^ and containing 42 sampling points, each corresponding to an area of 0.0052 mm^2^. Consecutive fields were evaluated across the entire lobe. The volume fraction of fibrosis was expressed as the percentage of sampling points overlying fibrotic tissue relative to the total number of points assessed. The parenchymal area assessed per lung ranged from 28.0 mm^2^ to 136.0 mm^2^. Stained slides were also scanned using a NanoZoomer S60 (Hamamatsu Photonics K.K., Shizuoka, Japan). Images were imported into the Visiopharm Integrator System (VIS; version.2017.2.4.3387), and the fibrotic tissue area was quantified using an automated VIS Analysis Protocol Package.

### 4.6. Assessment of Biomarkers

Frozen right lung lobes were used for biomarker analysis as previously described [12]. Briefly, tissue samples were homogenized in ice-cold 1x PBS (10010023, Thermo Fisher Scientific, Waltham, MA, USA) in a ratio of 1:10 (*w*/*v*), using a gentleMACS™ Dissociator (Miltenyi Biotec, Bergisch Gladbach, Germany) followed by a Polytron PT2500E (Kinematica, Malters, Switzerland) in the presence of protease and phosphatase inhibitors (PPC2020, Sigma-Aldrich, Saint Louis, MO, USA). Homogenates were subsequently clarified by centrifugation, and the resulting supernatants were used for biochemical measurements. The biomarker panel included: procollagen-I, WISP-1, KL-6 (MUC-1) and MMP7 as markers of fibrosis and epithelial injury and VEGF and FGF2 as target engagement readouts associated with nintedanib’s mechanism of action, as previously described [12]. All markers were quantified by enzyme-linked immunosorbent assay (ELISA) commercial kits, procollagen-I (ab210579, Abcam, Cambridge, UK), WISP-1 (Mouse/Rat WISP-1/CCN4 Quantikine ELISA Kit, MWSP10, Bio-Techne, Minneapolis, MN, USA), MUC 1/KL-6 (Rat MUC1 ELISA Kit, Novus Biologicals, Bio-Techne, Minneapolis, MN, USA), MMP7 (Rat MMP-7 ELISA Kit, NBP3-06896, Bio-Techne, Minneapolis, MN, USA), FGF2 (Mouse and Rat FGF basic/FGF2/bFGF ELISA Kit—Quantikine, MFB00, Bio-Techne, Minneapolis, MN, USA) and VEGF-A (Rat VEGF-A ELISA Kit, ab100787, Abcam, Cambridge, UK), in accordance with the supplier’s instructions. Marker concentrations were initially obtained as picograms (pg)/mL of lung homogenate supernatant, corrected for the assay dilution factor. The total amount of each biomarker was estimated by multiplying the measured concentration by the corresponding right lung weight. The final results were expressed as pg in the right lung as previously described [12].

### 4.7. Statistical Analysis

For food consumption, body and lung weight, histological and biomarker analyses, an individual animal was considered the experimental unit.

Statistical analysis was performed using Graph-Pad Prism, 10.1.0 software version for Windows (Graph-Pad Software, San Diego, CA, USA). All data were reported as the mean ± SEM except for the nintedanib plasma level concentration expressed as the mean ± SD. Comparisons between BLM-treated animals and corresponding vehicles were carried out using a one-way ANOVA followed by Dunnett’s test. A *p*-value ≤ 0.05 was considered statistically significant.

## Figures and Tables

**Table 1:** Nintedanib content in medicated chow pellets quantified by LC–MS.

Sample	mg API	mg Pellet	% *w*/*w*
Pellet 1	5.71	3404.34	0.168
Pellet 2	5.51	3610.78	0.153
Pellet 3	5.99	3635.87	0.165
Pellet 4	6.34	3694.63	0.171
Pellet 5	5.93	3606.15	0.164
Pellet 6	5.68	3733.20	0.152
Pellet 7	5.17	3116.82	0.166
Pellet 8	5.05	3074.41	0.164
Pellet 9	5.48	3341.41	0.164
Pellet 10	5.19	3211.77	0.162
Pellet 11	6.49	3860.90	0.168
Pellet 12	5.44	3480.78	0.156
Pellet 13	5.19	3044.63	0.170
Pellet 14	5.87	3565.50	0.165
Mean			0.163
RSD%			3.65

## Data Availability

The original contributions presented in this study are included in the article. Further inquiries can be directed to the corresponding author.

## Supplementary Materials

The following supporting information can be downloaded at: https://www.mdpi.com/article/10.3390/ijms27188158/s1.
