# Supplementary material for: A Comparative Assessment of the Antifibrotic Effect of Nintedanib Administered via a Medicated Diet or Oral Gavage in a Rat Model of Bleomycin-Induced Pulmonary Fibrosis
Source: Int J Mol Sci. 2026 Sep 13;27(18):8158. doi: 10.3390/ijms27188158 (PMC13607681; doi:10.3390/ijms27188158)
Supplement: Supplementary file 1 [file ijms-27-08158-s001.zip › ijms-4497406-supplementary.pdf]

Supplementary materials

LC–MS/MRM conditions for nintedanib quantification in medicated chow

LC–MS/MRM analysis was performed using the conditions summarized below.

Supplementary Table S1

**LC parameter**	**Condition**
UHPLC instrument	Waters Acquity
Column	Kinetex C8, 100 × 2.1 mm, 1.7 µm (Phenomenex)
Column temperature	50°C
Mobile phases	HCOONH4 0.025 M, pH 3 (A); acetonitrile + 0.1% formic acid (B)
Flow rate	0.5 mL/min
Stop time	5 min
UV detection	DAD
UV acquisition range	210–400 nm
Injection volume	5 µL
Sample solvent	Acetonitrile/water 60/40

LC parameters used for nintedanib analysis by UHPLC.

Supplementary Table S2

**Time (min)**	**%A**	**%B**
0.00	99.0	1.0
3.00	20.0	80.0
3.50	20.0	80.0
4.00	99.0	1.0
5.00	99.0	1.0

LC profile showing the relative percentages of mobile phase A and mobile phase B at each time point.

Supplementary Table S3

**MS parameter**	**Full-scan MS scan, ES+**	**Full-scan MS scan, ES–**	**MRM, ES+**
MS instrument	Waters XEVO-TQS		
Acquisition mode	Full-scan MS scan	Full-scan MS scan	MRM
Polarity	ES+	ES–	ES+
Capillary	1.20 kV	1.20 kV	1.20 kV
Cone	10.0 V	10.0 V	10 V
Source offset	60.0 V	60.0 V	60 V

Source temperature	150°C	150°C	150°C
Desolvation temperature	250°C	250°C	500°C
Cone gas flow	250 L/h	250 L/h	250 L/h
Desolvation gas flow	500 L/h	500 L/h	500 L/h
Scan duration	0.15 s	0.15 s	—
Interscan delay	0.003 s	0.003 s	—
Mass range	150–1000	150–1000	—
Parent ion	—	—	m/z 540.27
Daughter ions	—	—	m/z 70.10; 113.06
Dwell time	—	—	0.025 s
MRM cone	—	—	74 V
Collision energy	—	—	36; 26 V

MS and MRM acquisition parameters used for nintedanib detection.

Supplementary Figure S1

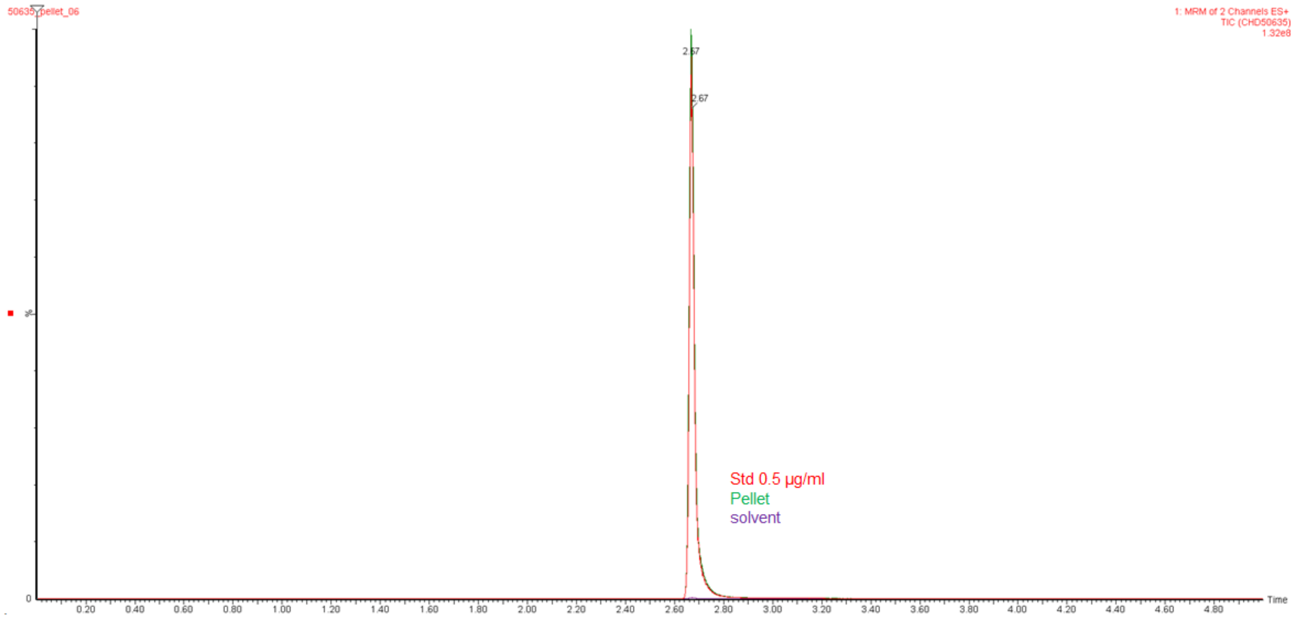

LC/MS (MRM acquisition) chromatograms of nintedanib in standard and in pellet.

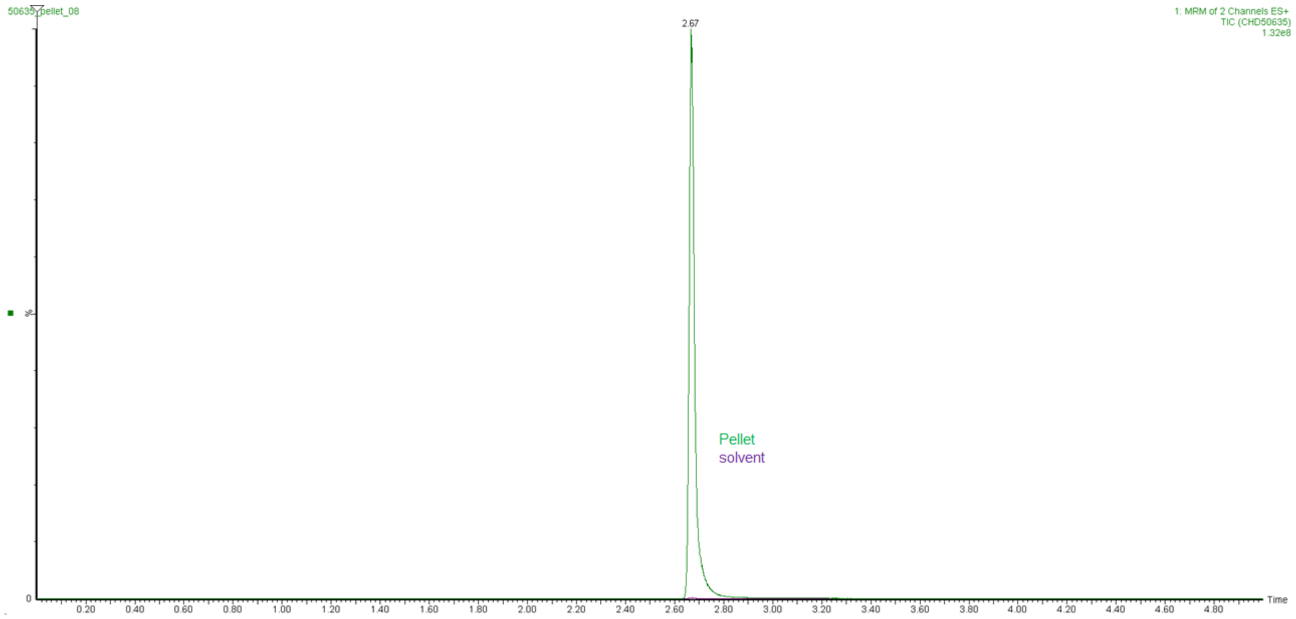

LC/MS (MRM acquisition) chromatograms of nintedanib in pellet.

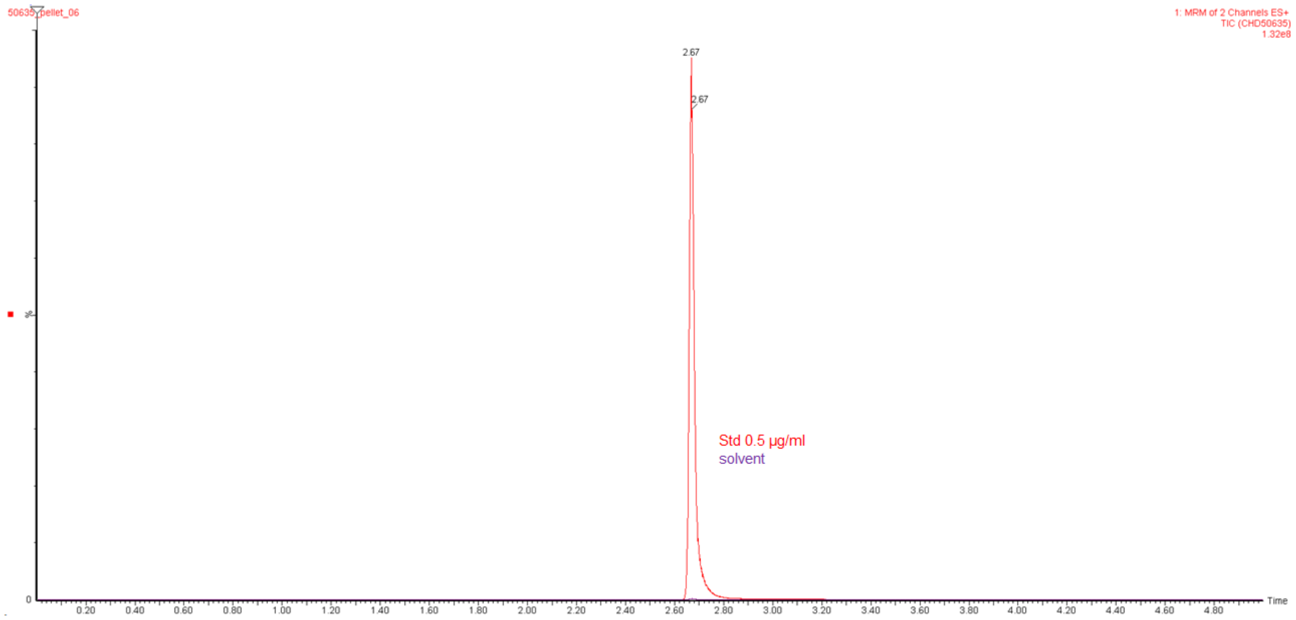

LC/MS (MRM acquisition) chromatograms of nintedanib in standard.

Supplementary Table S4

Estimated individual nintedanib intake during administration via medicated chow

Animal	Day 9	Day 11	Day14	Day 16	Day 18	Day 22	Day 24	Day 28
**Nint 1**	105	112	115	103	98	80	81	93
**Nint 2**	110	121	124	112	103	82	85	100
**Nint 3**	114	128	123	106	95	74	76	84
**Nint 4**	90	108	115	127	131	114	101	97
**Nint 5**	107	121	126	135	143	117	106	104
**Nint 6**	77	87	93	104	111	99	91	91
**Nint 7**	112	132	161	166	169	151	130	128
**Nint 8**	89	95	100	101	104	96	86	86
**Nint 9**	101	103	109	111	112	107	95	96

The table reports the estimated nintedanib dose received by each animal during the treatment period, calculated at each food-consumption monitoring interval from the amount of medicated chow consumed. Nominal nintedanib concentration in the diet was 1.6 g/kg chow. Values are expressed as mg/kg/day of nintedanib. These data integrate the graph shown in Figure 1b by reporting inter-animal and temporal variability in nintedanib intake.
